# Supplementary material for: Single cell analysis reveals immune cell–adipocyte crosstalk regulating the transcription of thermogenic adipocytes
Source: eLife. 2019 Oct 23;8:e49501. doi: 10.7554/eLife.49501 (PMC6837845; doi:10.7554/eLife.49501)
Supplement: Supplementary file 2. [file elife-49501-supp2.docx]

**TABLE S2. QPCR PRIMERS USED IN THE STUDY**

| **PRIMER** | **FORWARD** | **REVERSE** |
| --- | --- | --- |
| UCP1 | GGCCTCTACGACTCAGTCCA | TAAGCCGGCTGAGATCTTGT |
| CIDEA | AGCCACCAACATCACCAAT | CGACTTGACCCGACCTTG |
| ADRB3 | TCTTGGGGCAACCAGTCAAG | CACCGCTCAACAGGTTTGATG |
| ELOVL3 | TCCGCGTTCTCATGTAGGTCT | GGACCTGATGCAACCCTATGA |
| COX8B | GAACCATGAAGCCAACGACT | GCGAAGTTCACAGTGGTTCC |
| PRDM16 | GCCATGTGTCAGATCAACGA | CCTTCTTTCACATGCACCAA |
| OTOP1 | GACAACCCGATGTCTGGACT | GCCAAAGCAAATTTCCTCCA |
| SLC27A2 | GCGTGCCTCAACTACAACATT | CCTCCTCCACAGCTTCTTGT |
| ELOVL6 | CAGCAAAGCACCCGAACTA | AGGAGCACAGTGATGTGGTG |
| EPHX1 | TCCCTCAATTCCTGGCTATG | GGCCACCGAATTTAAACCTT |
| ADIPOQ | CCGGAACCCCTGGCAG | CTGAACGCTGAGCGATACACA |
| FABP4 | TTCGATGAAATGACCGCAGA | GGTCGACTTTCCATCCCACTT |
| PLIN1 | CCATCTCTACCCGCCTTCG | CTTGTCAGAGGTGCTTGCAATG |
| AQP7 | CTGGATGAGGCATTCGTGACT | TGATGGCGAAGAGACACAGC |
| TLE3 | AGTCTCGCCTCCATTCCTG | CATCTGCCCATCAGCACTC |
| PPARγ | AACTCTGGGAGATTCTCCTGTTGA | TGGTAATTTCTTGTGAAGTGCTCATA |
| DIO2 | CAGTGTGGTGCACGTCTCCAATC | TGAACCAAAGTTGACCACCAG |
| TLE3 | TGGTGAGCTTTGGAGCTGTT | CGGTTTCCCTCCAGGAAT |
| EVA1 | GTC CCA ACC AGA CCA TCA AC | CTC CAT CTT GCT CTG GAA GC |
| EAR2 | CCTGTAACCCCAGAACTCCA | CAGATGAGCAAAGGTGCAAA |
| PM20D1 | TGGAGAATATATCCGCAAAGC | GCCACGACTTCATGTTGGA |
| PPARGC1α | GAAAAGGCCAAACAGAGAGA | GTAAATCACACGGCGCTCTT |
| 36B4 | AGATGCAGCAGATCCGCAT | GTTCTTGCCCATCAGCACC |
